# Violence narratives of Mexican women treated in mutual-aid residential centers for addiction treatment

**DOI:** 10.1186/s13011-016-0083-0

**Published:** 2016-11-30

**Authors:** Ignacio Lozano-Verduzco, Martha Romero-Mendoza, Rodrigo Marín-Navarrete

**Affiliations:** 1 Universidad Pedagógica Nacional, Ciudad de México, Mexico; 2 Calz. Instituto Nacional de Psiquiatría Ramón de la Fuente Muñiz, Calz. México-Xochimilco 101, Tlalpan, Huipulco, 14370 Ciudad de México, Mexico; 3 Universidad Iberoamericana, Ciudad de México, Mexico

**Keywords:** Addiction, Alcohol and drug abuse, Violence, Addiction treatment, Gender

## Abstract

**Background:**

Violence against women is a social and public health issue in Mexico. The aim of this article is to explore violence among an understudied group of women, who attended Mutual-Aid Residential Centers for Addiction Treatment and experienced stigma both as women and addicts. These centers are particular kind of addiction treatment services that stem from 12-step philosophy, but that have been found to manipulate said philosophy and exercise extreme forms of psychological and physical violence.

**Methods:**

Thirteen semi-structured interviews were carried in 2014 and 2015 out with women who resided in at least one of these centers to understand their experiences of violence prior and during their rehabilitation process. The interview guide covered questions regarding substance use initiations, family violence and dynamics, and rehabilitation experiences. Qualitative data was analyzed using interpretative-phenomenological analysis.

**Results:**

Two categories emerged: violence and substance use and abuse, and violence against women in recovery. Results show that all participants experienced violence in their family since childhood, particularly sexual and physical violence. As a result, participants experienced guilt, sadness and shame, which led them to contexts of consumption. Violence continued as they explored alcohol and drug use, even though women felt empowered.

**Conclusions:**

Treatment reproduced masculine violence constantly, but women felt that they were in a context that helped them understand their addiction. Even though women felt these centers played a crucial role in their recovery, women’s particular needs and experiences are not considered in the treatment program.

## Background

Violence against women and drug abuse are considered a public health and social, national problems in Mexico [1, 2]. According to the National Institute of Women, 47% of women reported being victims of violence. The most common type of violence reported was emotional violence, followed by economic, physical, and sexual violence. Surveys also show that women are victims of violence outside their home: in 2011, 22.5% of women reported having been victims of violence in the last year in a public space (workplace, school or their community). In all cases, men were the perpetrators of this violence [3].

Substance abuse is the main risk factor for lost healthy years for young Mexican women ages 15 to 24; drug use is the ninth cause of YLDs, the twelfth cause of DALYs, and the fourth cause of premature death for women 15 to 49 [1]. According to Romero’s [4] revision of addiction literature in Mexico, women’s risk of violence increases with drug use and abuse.

Women’s risk of violence increases with drug use and abuse [4]. Literature shows that health and social consequences due to substance use and abuse affect women more than they do men. This is because of stigma and syndemics associated with women who use alcohol and drugs, and because health systems do not cover all of women’s n [4, 5]. This can be considered a form of structural violence against women. Structural violence must be differentiated from interpersonal violence (such as physical, sexual and emotional). The former refers to the way in which a social structure or institution prevents individuals from meeting their basic needs, and gender violence must be considered as a form of structural violence [6]. Physical, sexual, emotional and economic violence are considered forms of interpersonal violence, which according to the Mexican General Law for Women to Access a Life Free of Violence [7] states that these types of violence are constituted by any act or omission that locates women in any form of danger, that attempts against their human dignity, wellbeing, and mental and physical health, and that affects their survival. Both types of violence reproduce each other constantly; symbolic violence allows the existence of interpersonal violence where women are victims, and interpersonal violence feeds institutions that practice structural violence.

Violence is strongly associated to substance use among women. Macy et al. [8] found that use substances have an important history of gender violence that often starts with the first episode of substance use. Other study reported that women who alcohol and cocaine abuse predicts any kind of intimate partner violence [9]. Romero et al. [10] found that over 70% of the women with substance use disorders have experienced intimate partner violence and suffered sexual abuse in their childhood.

On the other hand, some women consider drug and alcohol use as a form of liberating themselves from that social system, which produces emotions such as joy and happiness. Substance use is also lived as a way of escaping from everyday problems. However, as use turns into abuse, these emotions transform into guilt, because women felt they were harming their families and immediate contexts [11].

The lack of addiction treatment in Mexico led to the formation of different forms of social organization of non-profit organizations and clinics to treat addiction problems [12, 13]. Many of these organizations in Mexico, denominated by Marín-Navarrete et al. [14] as mutual-aid residential centers for addiction treatment (CRAMAA, for its acronym in Spanish), have based their interventions on interpretations of the twelve-step philosophy that gained popularity during the 80’s and 90’s [15]. These groups began to transform into centers that provided meetings 24 h a day, and later into residential centers for addiction treatment [15]. However, most of these centers operate outside the law, and hold their residents against their will [14, 16] and suffer from constant forms of violence [14, 16–18]. The majority of people treated in these centers are men, and thus, research has been centered on them [19], and has invisibilized women’s experience in recovery processes in CRAMAA. However, the little research shows that between 59 and 72% of women in CRAMAA present co-occurring disorders [20, 21].

CRAMAAs interventions are based on the 12-step philosophy and are very heterogeneous in their recovery model. It has been found that residence in these centers can vary from 60 days to a whole year. Notwithstanding, what seems clear that in general, the persons in charge of the CRAMAAs are the sponsors with the most amount of time in sobriety [14, 16, 17].

This data makes it clear that women face violence due to gender reasons and are stigmatized because they are substance users and abusers. This intersection helps introduce a syndemic framework to analyze violence against women in a rehabilitation community with cultural particularities [22]. Ostrach and Singer [23] identify that factors such as the structures of gender relations, sexual power dynamics, the feminization of poverty, gender-based violence, and the nature of economic survival strategies available to women interact with social elements to create risky pathways for women. With this in mind, this study presents the primary objective of understanding the different forms of violence women who use and abuse substances experience throughout their life stories.

## Method

Considering women’s vulnerable condition in Mexico, and their double stigma as substance users, the research team became interested in analyzing women’s experiences of violence in relationship to their substance abuse. Specifically, this research centers on women’s lived experiences as victims of gender violence and the conflation it has with substance use. This led the research team to dissect the primary aim into three specific objectives. The first: to analyze the different forms of violence women experienced that led them to substance use. The second: to analyze women’s experiences of violence during their substance use and abuse. And the third, to analyze structural and interpersonal violence against women once they initiated a recovery process in CRAMAAs. This involved analyzing family violence, partner violence, peer violence and violence within rehabilitation contexts. Researchers and clinicians with expertise in addiction and gender violence integrated the research team.

### Participants and procedure

The first contact with the participants was through a private clinic specialized in addiction treatment in Mexico City. Afterwards, women were recruited through a convenience snowball sample technique [24]. Because there is no prior research on the topic of women treated in CRAMAAs, women were eligible to participate only if they had resided at least once in a CRAMAAs. Eligible women were contacted by the office manager of the private hospital who explained the objectives and procedures of the research project, emphasizing their anonymity and confidentiality, and that their participation was voluntary and would not interfere with the treatment they were receiving at the time. If women accepted to participate, they were scheduled to meet the female interviewer at a convenient day and time. A semi-structured focalized interview guide was prepared prior to the interviews. This guide covered topics such as, history of substance use and abuse, violence, stigma and rehabilitation, and experiences in CRAMAA. All interviews lasted between 85 and 135 min, were held in the addiction treatment clinic, were audio-recorded and transcribed verbatim in Spanish. The thirteen participants’ clinical and sociodemographic data are detailed in Table 1.

**Table 1:** Characteristics of participants

Participant	Age (years)	Number of times in CRAMAA’s	Substance of preference	First use	Marital status	Children	Employed	Monthly income (USD)	Comorbidity	Violence in childhood
M1	31	3	Alcohol	12	Single	No	Yes	400 (dependent on family)	None	Physical and psychological violence from mother
M2	27	3	Alcohol and marihuana	17	Single	No	Yes	300: waitress	None	Physical and psychological violence from parents
M3	25	4	Alcohol and cocaine	8	Single	No	Yes	350; NGO employee	Borderline Personality Disorder	Sexual abuse from peers
M4	25	2	Alcohol, marihuana, cocaine and amphetamines	19	Single	No	Yes	280; employee	none	Verbal violence from parents
M5	27	2	Alcohol anxiolytics	10	Single	No	No	800 (dependent on family; resided in a CRAMAA at the time of interview)	Schizophrenia	Negligence and abandonment
M6	35	1	Alcohol and cocaine	15	Single	No	Yes	2000: realtor	None	No
M7	22	2	Alcohol, marihuana and cocaine	14	Single	No	Yes	5200: teacher’s assistant	Borderline Personality Disorder	Psychological violence, negligence and abandonment from parents
M8	20	3	Alcohol, marihuana and amphetamines	13	Single	No	No	800 (dependent on family)	Borderline Personality Disorder	Physical and psychological violence from alcoholic father
M9	55	1	Alcohol	8	Divorced	Two children	No	4000; business owner	Yes, doesn’t remember the diagnosis	No
M10	23	3	Amphetamines	14	Single	No	No	600 (dependent on family)	Borderline Personality Disorder	Physical and psychological violence from mother; sexual violence from cousin
M11	29	1	Alcohol	14	Single	No	Yes	800; assistant	Borderline Personality Disorder	Negligence from mother
M12	30	1	Alcohol	18	Single	No	No	550; researcher	Major Depressive disorder	No
M13	18	1	Alcohol, marihuana	12	Single	No	No	None	Yes, doesn’t remember the diagnosis	Sexual harassment from family member, psychological and physical violence from parents

### Data analysis

The research project used interpretative-phenomenological analysis (IPA) [25, 26], which entailed a constant reading and re-reading of all the data. IPA suggests that all experiences are mediated by social and discursive representations that express both subjective and cultural aspects of the studied phenomenon. It suggests that it is possible to understand the phenomenon only through the respect of the participants’ narrations and the researcher’s interpretations of the narrations, achieved with the iterative process of reading and re-reading the transcripts. During this process, it became clear that eliciting sequential narratives from participants was problematic due to their difficulty in recalling violent events, especially those that occurred when they were under the influence of a substance. In some cases, these narratives were also result of a particular comorbidity.

The research team held meetings to discuss interpretations during each phase of analysis, as suggested by Smith and Osborne [26]. These authors suggest reading and re-reading transcripts in order to identify main themes. Initial suggestions of themes made by the first author were discussed with the second and third author until agreement. Second, the research team searched for connection between themes through the construction of clusters. Finally, the research team decided on “master themes” that they considered appeared throughout the transcripts. Each master theme was constituted of “secondary themes” that made a coherent whole without the necessity of appearing in al transcripts. The interpretations made by male authors were at times questioned and enriched by the female authors and the interviewer herself, allowing a richer and holistic comprehension of participants’ experiences. On other occasions, very medical interpretations emerged from clinicians that were richly discussed in order to provide historical and sociocultural context to those interpretations. The data presented in this article describes one of the emerging categories “stigma, violence, substance use and abuse”. Even though contacting eligible participants was quite difficult, the category reached saturation with 12 interviews [27, 28].

## Results

Ten of the participants had received psychiatric and psychological treatment in a private clinic in Mexico City, meaning that almost all of the participants had access to private medical services, and came from a middle-class socioeconomic background. Nearly all of them had suffered some sort of violent experience in their childhood, and nearly all of them were diagnosed with another disorder besides alcohol/drug abuse by a psychiatrist.

Intersectionality was also used during the analysis. According to this perspective, in order to undertake a fuller understanding of women’s experience, the overlapping of social identities must be central in the qualitative analysis [29]. Participants in this study were not only understood as women and thus in permanent potential of being victims of violence at the hands of men, but also as middle-class and “addicted” women.

When the participants were asked directly if they believed they had experienced some sort of violence in their life, one third of them answered no. When their narratives were explored more deeply, the analysis showed that all participants had lived some sort of violence as victims throughout their lives. The following excerpts serve as an introduction to show participant’s perceptions and reactions to the constant forms of direct and structural violence they received.

*“For example, they say that we women are always suffering from emotional crisis, but they say it’s our psychiatric problem. I mean, ugh! That makes me so angry! Sometimes I even want to kill them, I mean, if I’m having my period, I’m afraid, I’m cold, I want a coffee, I want to sleep, and it’s ‘you’re psychiatric crisis’, it’s an ‘episode’, they say. And I’m like, what? How? …” (M3, 25 years old; comorbid condition with Borderline Personality Disorder).*

*“Women are so much more stigmatized, like, a super drunk woman, with her dress falling off, her mascara running is seen as a whore, but a man is just a drunk.” (M1, 31 years old; no comorbid condition).*

*“I can feel the stigma, I wouldn’t be so singled out if I were a man. Since you are a women, you carry the responsibility of always looking pretty, always being the good girl. You have to carry that, being the drug addict” “M10, 23 years old; comorbid condition with Borderline Personality Disorder).*

Participants identify actions and discourses that stigmatize them: they show that women’s expression of emotions are understood as a crisis and problematic for traditional psychiatry, that their substance use constructs them as sexual objects rather than human subjects, and feel minimized by men when they drank.

### Victims of violence and substance abuse

Participants reported being victims of different forms of violence since before they started using drugs and alcohol; from male peers who tried to sexually abuse them, to parents yelling and insulting them, to living episodes of abandonment. Participants said that they did not immediately say anything to anyone about the violent episode because they felt as in a state of shock that did not allow them to speak up. Some participants spoke to their mothers years after the violent episode, who usually responded by minimizing the event or denying that it ever happened.

*“I was like eleven. And I told my friend ‘yes, I’ll do anything you want’. And she said, ‘get on your knees’ and she started humiliating me in front of them. And fear invaded me. She just said ‘shut up and get on your knees!’ And I didn’t do anything. And then she said: ‘Now you’re going to blow those guys’, and I got really scared. But I just spazzed out…blocked. And I just did it.” (M3, 25 years old; comorbid condition with Borderline Personality Disorder).*

*“…They just told me that my mom was going on vacation, and she never came back. I lived with my grandparents and I always asked them ‘when is she coming back?’ And it was sad because I felt even more alone…” (M1, 31 years old; no comorbid condition).*

Participants’ experiences show that on occasions, women are also de perpetrators of violence against women. In this case, a friend motivated the sexual abuse from other male teenagers. This data is important, but it does not mean that women as a group or social class are violent with themselves, but that on occasions, they occupy masculine subject positions [30].

*“It was unreal that everything was ok, I started gaining weight for no reason, I didn’t understand why, or why I would cry all night. I felt really sad and alone. Nobody was ever around, nobody. My dad even forbade me to see my mom. I couldn’t even talk to him on the phone, I couldn’t see her.” (M13, 18 years old; no comorbid condition).*

These episodes of violence also produced feelings of sadness, fear, guilt, and loneliness among participants. Women reported feeling “weird” and “out of place” among their families and peers, but that they felt welcomed by those who already used substances. When women first used substances, they felt empowered, so they continued their use. However, being victims of family violence produced an almost permanent feeling of emotional discomfort, which in turn motivated them to use — as using produced empowerment —, or allowed them to temporarily forget their discomfort all together. More importantly, substance use allowed participants to feel at the same social level as men, because they also felt accompanied and part of a reference group.

*“…At first I liked being a drug addict, because in my trip, I felt that I was the best, it was like ‘Oh! That girl drinks six liters of pulque,* 1 *smokes four joints’. I mean, you believe you’re almighty. It made me feel good…” (M4, 25 years old; no comorbid condition).*

These positive feelings were fundamental for women to continue their use [31, 32], establishing a clear connection between violence and substance use. Once part of a culture of consumption, the access to the substance was easy and became part of daily life.

Some women reported feeling “lonely” and “empty” after using and during their hangover. This also led them to use again. This escalated form of using turned into abuse, and women identified it as “disastrous”:

*“…Everything became a disaster…My daily schedule, waking up, eating, sleeping, my family relationships (tears), friendships, with my boyfriend…” (M1, 31 years old; no comorbid condition).*

This extract reflects the bio-socio-psychological degradation that occurs when use turns into abuse, as well as the emotional discomfort that this degradation produces. The increase in use and abuse of any substance made it more likely for women to suffer from gender violence once again. Participants reported that their male partner would threaten, blackmail, beat, minimize, insult and/or yell at them, using their substance abuse as a form of discrimination and stigma.

*“But he (my boyfriend) knew how to manipulate me really well… he always gave me the drug so I would feel bad, because I never felt good… He thought he was the man of the house, but it was my house and my money, but the fact that he controlled the drug gave him power…” (M6, 35 years old; no comorbid condition).*

*“Anyway, I think because of the people I started to hang out with, I felt alone, and bleak. I think it was because of the people, my boyfriends…” (M10, 23 years old; comorbid condition with Borderline Personality Disorder).*

Women reported a sense of losing autonomy and clarity in their relationship because of drug use, as well as taken advantage of by their IMP. During intoxication, women were exposed to violence from other perpetrators, such as friends or strangers. Some women reported having been sexually abused or raped without them being conscious of the event:

*“… I remember we were drinking on the street. I lost it and I wake up in a horrible, ugly room, still dizzy from the drinking. I stand up and I started to see blood. When I went to the bathroom, I saw a puncture beside the labia, the guy punctured me with something…It was like four centimeters wide and two in depth. It really hurt…” (M3, 25 years old; comorbid condition with Borderline Personality Disorder).*

*“I always heard ‘I’ll just get her drunk and get her in bed’… like I was just good for sex…When my boyfriend and I had sex he would slap me, at first it caught me by surprise, but I didn’t’ say anything, but I didn’t like it, I thought I was exaggerating. But it kept happening, and I thought to myself ‘it doesn’t matter, I really like this guy, and he’s nice’. There were times when I thought he would punch me…” (M11, 23 years old; comorbid condition with Borderline Personality Disorder).*

However, the use and abuse of drugs and alcohol that mitigated emotional discomfort opened the door for further episodes of violence. Women felt afraid after such violent episodes, a feeling that did not allow them to speak up and report the violence they had been victims of. This fear was also a product of structural violence that stems from gender culture.

Some women reported losing an emotional connection with the reality they lived. Participants reported that living these experiences of violence led them to feel extremely insecure in many ways: they weren’t comfortable with the way they looked, it was difficult for them to trust anyone, they felt paranoid, and even had difficulty looking at themselves in the mirror. These feelings led them directly to more use of drugs. Thus, violence against women is crucial in understanding motivation to use and abuse any substance. Participants are not only victims of gender violence, but their substance abuse intersects with their gender condition to produce a more subordinated subject as women abusers.

### Violence against women in Mutual-Aid Centers for Addiction Treatment

Participants did not want to be admitted into a CRAMAA, and some of them were led there with lies, fraud and against their will. The immediate family actively participated in convincing the participants to admit themselves into a CRAMAA. Participants reported important events of violence within the CRAMAAs, from sexual harassment, to insults and humiliation:

*“On the tribune…they constantly threaten you, and say stuff like ‘look at you, you’re a whore’, they call you ‘asshole’, ‘addict’, ‘crazy’…The ones that have a psychiatric diagnosis suffer the most, the violence against them is worse…” (M5, 27 years old; comorbid condition with Schizophrenia).*

*“He would lock me up in the dining room, or stuff like that, just because he liked locking me up” (M5, 27 years old; comorbid condition with Schizophrenia).*

All participants reported that violence was used as a technique for recovery within CRAMAAs, mostly from the sponsor’s part. In particular, they spoke of the use of the tribune and a technique they named “feedback” as the most violent experiences. Feedback consisted of hearing other people’s opinion of them without them having the opportunity to speak up or defend themselves. Some women also referred that women with psychiatric comorbidity were further stigmatized: as women, substance abusers and “crazies”.

*“It’s outright disrespectful that men can go on the tribune and say ‘women are for fucking’. That made me really angry. So men can say anything they want, but as a woman you can’t say anything about sex because that’s inviting men to think about sex, but at the same time, it’s minimizing and having control over women…” (M11, 29 years old; comorbid condition with Borderline Personality Disorder)*

*“If you turned around they would slap you in the face. I know some sponsors had sex with the girls…One day they locked us up for three days in a room…No bathroom, no bucket to pee or take a crap, no food…” (M5, 27 years old; comorbid condition with Schizophrenia).*

*“I was saying goodbye to my friend…But they caught us (sleeping together) and they took me to the sponsor—I had one foot out the door—and the sponsor said ‘fifteen more days’ (as punishment for sleeping with her friend)…All they give you is a simple broth with no salt, what no one eats of the chicken, that’ what they feed you. Slept in a tiny room with dirty, smelly covers, used a bucket as a toilet, took baths at six in the morning with freezing water, we had to sit on a wooden board for a chair, looking straight ahead, we couldn’t smoke, we had to wipe ourselves with newspaper when we went to the bathroom…” (M10, 23 years old; comorbid condition with Borderline Personality Disorder).*

Women were victims of different forms of violence at the hands of men: structural gender violence expressed in phrases such as “women are for fucking”, to direct physical and sexual violence when acting “out of order” or simply because male residents and sponsors saw them as sexual objects, or subhuman because they abused substances.

On occasions participants also felt violated by other women within CRAMAAs. Participants described the women who were violent towards them as “butch”, “lesbians” and “macho”. The image of a masculine woman was in itself, perceived as threatening, or equivalent to manhood. It’s not really the male body that seems to be violent, but the position of power that that body occupies through practices and acts that are considered masculine.

*“…If (in the CRAMAAs) there are only women, it’s like they’re men. There’s that aggressiveness and a lot of them are lesbians. In those places you need to watch out that they don’t steal from you” (M2, 27 years old; no comorbid condition).*

Women found it difficult to build significant relationships within CRAMAAs, and usually did so with people that was also in a subordinated position to masculinity. These relationships were central to women’s recovery.

*“I was really close to one of the sponsors. He was nice and provided confidence because he was gay, so I knew it was alright with him…He would hold me and I would cry…” (M7, 22 years old; comorbid condition with Borderline Personality Disorder).*

When women feel supported and comprehended, they are able to reflect on their stories of substance use and abuse, and may understand it from a different point of view. This is achieved once women feel part of the particular group of women who also reside in CRAMAAs and are able to build a collective identity within that group. With this achievement, women felt sisterhood and affect that allowed the production of emotional wellbeing that keeps them distanced from further substance abuse.

## Discussions

As a whole, participants’ narrations reveal that not all of them understand what violence is, that gender culture produces hierarchies between men and women, and that it is women’s bodies that are subordinated to men’s, but that not all women are conscious of this gender condition, especially the ones who felt indignation and anger. Not understanding these social and personal conditions made it difficult for women to identify acts of violence, report them and try to prevent them. Other women also violated participants, women that occupied a position of power in a masculine subject position [30]. Since gender culture socializes its norms on all individuals, women can also become perpetrators of violence under certain conditions.

Violence against women perpetrated by men is part of a social and public health issue that has been addressed globally [33]. We must understand not only macro contexts and structures, but also, how these structures impact on smaller communities, such as the one described in this text. Even though women as a social category share many elements, such as male subordination, there are also other characteristics that differentiate between groups of women. One of these characteristics is sexual orientation. One of the participants reported identifying herself as a lesbian, it is important to consider homosexuality as a characteristic that requires particular attention within the health system and CRAMAAs due to the fact that their bodies are usually understood as “masculine”, and struggle through homophobia [34, 35], which can lead to the invisibilization of their desires and needs.

The experience of violence as a victim positioned women in a subordinated status to their aggressor. Thus, women came to understand that femininity was subjected to masculinity, and this acted as a catalyst for drug and alcohol use. Violence against women eliminates understanding women as autonomous and agentic subjects through this particular biopolitical syndemics [23, 36]. The experience of victimization and subordination produced feelings such as sadness, guilt and anger. These feelings, accompanied by the women’s incursion into cultures of consumption [31], led them into substance use. Women reported feeling euphoria, happiness or what one participant named “social anesthesia” while being intoxicated.

Consuming in certain contexts allowed participants to feel a sense of belonging and identity that they felt they lacked when sober. On one hand, women felt liberated and empowered when using substances. However, they were seen as usable objects for men when intoxicated. Their substance use breaks gender norms in their contexts, but at the same time, they are restricted by the very same norms when they are seen as objects subjected to masculine power.

Another condition that differentiates these participants from women in general, was their substance use and abuse. Women were exposed to other forms of violence from male friends and strangers. IMPs were usually the ones who had access to the substance, either because they bought it or dealt with it, thus controlling the women’s access to it, and in doing so, manipulated them into sexual practices that they were not comfortable performing (resulting in sexual violence), or demonstrated physical and psychological violence.

Regarding violence within CRAMAAs, data showed that rules and norms are extremely endorsed within these centers, on occasions, leading to subtle and explicit expressions of violence against women [15, 17], which generated fear, a fear that stopped them from speaking up regarding being victims of any sort of violence, or about things that made them feel uncomfortable. This keeping quiet not only allowed women to not be singled out or located in further positions of subordination, but it also allowed reinforcing feminine stereotypes as passive and docile. Such episodes of gender violence against women are also common in different parts of Latin America because of the widespread misogynist gender culture. Other forms of structural violence in CRAMAAs have been reported elsewhere: rotten food, physical torture and harassment [15–17]. However, women live through permanent forms of sexual violence that is impregnated in psychological and physical abuse expressed in CRAMAAs. In other words, women endure normalized psychological and physical violence with misogynist tones, as well as outright sexual violence within CRAMAAs.

Some forms of psychological and physical violence are endorsed as part of structural violence within residential centers. This helps understand that violence is part of recovery tactics on one hand, but that neither sponsors nor family members know how to treat women who require substance and/or psychiatric treatment. Violence is used as “punishment” for not complying with the centers’ rules, or 12-step philosophy, or because they are women. So instead, families and sponsors find ways to calm participants through different forms and expressions of gender violence. Another form of structural gender violence became evident when women were asked about their dual diagnosis. Even though women with comorbidity knew they had more than one psychiatric diagnosis, they did not understand what that meant; they were ignorant on their symptomatology and treatment, as well as how that affected their substance abuse.

Participants reported feeling angry, upset and frustrated after episodes of violence. Such emotions become dangerous for women because they are the ones that motivate relapses, craving and drug/alcohol use. Violence experienced by women is a form of objectifying them. Entering a recovery center to treat substance abuse where violence is constantly used, is also a form of re-victimizing women and repeating the violent episodes they were victims of prior to their recovery that initially escalated their consumption.

## Conclusions

The small sample of this study can be considered a limitation, because it shows a partial array of experiences. However, all categories reached saturation. Dealing with a hidden population in vulnerable conditions hinders the possibility of working with large samples. Furthermore, the amounts of women who use and abuse substances are considerably lower than men’s. There is also a strong stigma discriminating women who consume substances, which makes it difficult for them to seek treatment and volunteer for research participation. Because of this, and the detailed narratives provided by interviews, the data obtained is valuable in itself. Further research is recommended to deepen into women’s experiences of substance us and abuse in relationship to violence.

Participants’ experiences represent structural violence that locates women under masculine hegemony. Considering that participants felt defeated after these experiences may help understand the subordinated position they occupy as women. Because of this subordination, participants did not speak of those acts of violence to any adult immediately afterwards. The few women who did say something after the event turned to their mother, who minimized or denied such acts. The mother’s reaction may be understood as another form of violence, through omission, and thus contributing to the expansion of men’s hegemony over women because they collude with this hegemonic masculinity [37, 38].

Interviews also showed an intersection of social categories that increased women’s social vulnerability [36, 39]. Their status as consumers exposed them to other forms of violence, particularly sexual violence, perpetrated mostly by their IMP, and on occasions by male friends or strangers. Furthermore, this violence was also used upon them once they resided in CRAMAAs, where other social categories intersected with their identity: their psychiatric status and sexual orientation. Women with a psychiatric co-occurrence were at a greater risk from being victims of male violence in different forms. The syndemics of substance use and abuse shows that emotional consequences (particularly loneliness and sadness) of family violence in childhood and youth, in its intersection with biopolitical elements (such as unequal gender relations and objectification) help produce psychiatric comorbidity and further stigmatize women’s status, as well as introduce them to cultures of substance consumption. Experiences within CRAMAAs show that sexual violence is printed upon other forms of violent recovery tactics.

Health policy may greatly benefit from understanding these women’s experiences in order to reduce gender violence against women and thus its effects on emotions and substance use, but more particularly to pay close attention to the services offered by CRAMAAs, and how sponsors interpret the AA philosophy. Participant’s experiences show that sponsors try to lead residents intro recovery through power and violent relations that lead to the production of other negative feelings. Even though women’s experiences highlight negative aspects of CRAMAAs, we must consider that all of the participants were on a path to recovery, since they had not consumed alcohol or drugs since their last stay in a CRAMAAs. This fact is of the utmost relevance because it is an important indicator of rehabilitation. Results also show the importance of treating the consequences of being victims of violence parallel to substance abuse treatment. Women received treatment only for their substance abuse, but never for the violence they had experienced, its consequences, or their comorbidity status. Since women are already at risk of being victims of violence, great care must be considered when treating them in drug and alcohol rehabilitation. A context that stimulates power and violent relationships seems to do little for women’s recovery. We firmly suggest that CRAMAAs and other recovery centers consider women’s experiences of violence as important aspects in their lives and medical record, as well as elements that need to be introduced into health policy and therapeutic models for recovery.

## Footnotes

1 Pulque is a traditional mexican fermented drink with around 8% of alcohol
